# Supplementary material for: Organic Solar Cells Improved by Optically Resonant Silicon Nanoparticles
Source: Nanomaterials (Basel). 2022 Nov 6;12(21):3916. doi: 10.3390/nano12213916 (PMC9656450; doi:10.3390/nano12213916)
Supplement: Supplementary file 1 [file nanomaterials-12-03916-s001.zip › nanomaterials-1999570-supplementary.pdf]

Supplementary Materials

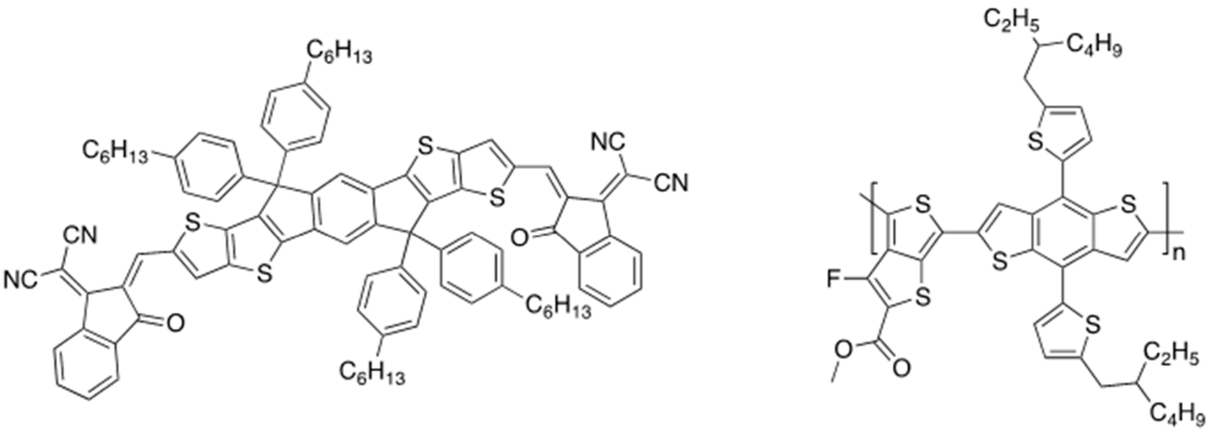

ITIC PTB7-Th

Figure S1. Chemical of nonfullerene acceptor ITIC (3,9-bis(2-methylene-(3-(1,1-dicyanomethylene)-indanone)- 5,5,11,11-tetrakis(4-hexylphenyl)- dithieno[2,3-d:2′,3′-d′]-s-indaceno[1,2-b:5,6-b′]-dithiophene) and repeat unit structures of donor polymer molecules PTB7-Th (PBDTTT-EFT, Poly-[4,8-bis-(5- (2-ethylhexyl)- thiophen-2-yl)benzo-[1,2-b;4,5-b']-dithiophene-2,6-diyl-alt-(4-(2--ethyl- hexyl)-3-fluorothieno- [3,4-b]thiophene-)-2-carboxylate-2-6-diyl)]) used in the study.

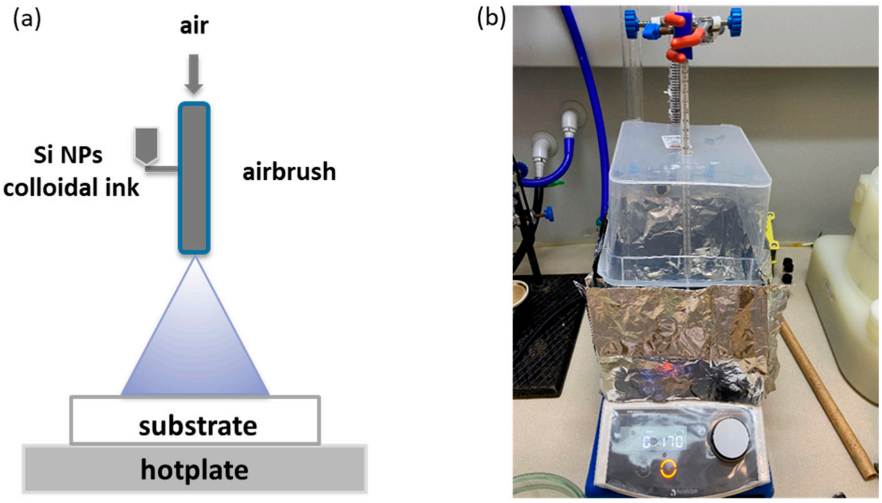

Figure S2. A schematic diagram of the spray coating technique (a) and photo of the setup (b).

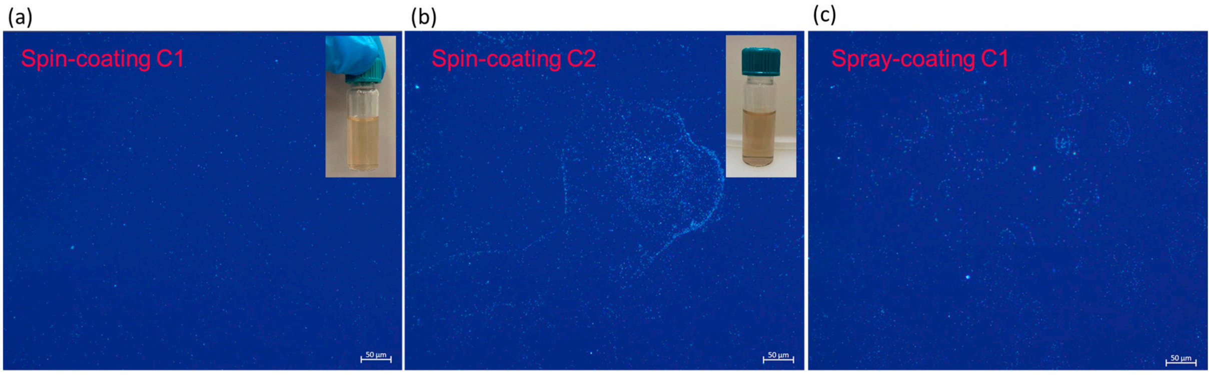

Figure S3. Optical images in the dark-field scheme of silicon nanoparticles deposited on ZnO interlayer by spin-coating and spray-coating techniques: (a) spin-coating of diluted colloidal Si NPs ink (C1); (b) spin-coating of ink with higher concentration of Si NPs (C2); (c) spray-coating of ink with concentration C1.

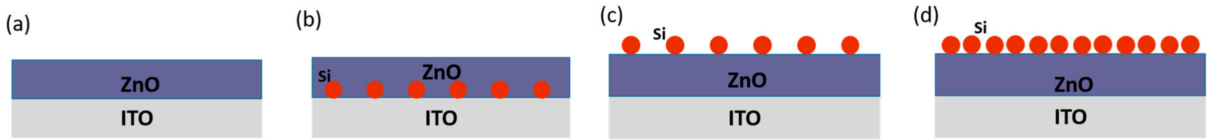

Figure S4. Schematic drawings of OPV devices without dielectric nanoparticles (a) and with dielectric Si nanoparticles incorporated in the buffer layer ZnO (b), c) Si NPs deposited ZnO/ITO substrates (ZnO/Si) with fewer concentrations (C1), and d) Si NPs deposited on ZnO/ITO substrate with higher concentrations (C2).

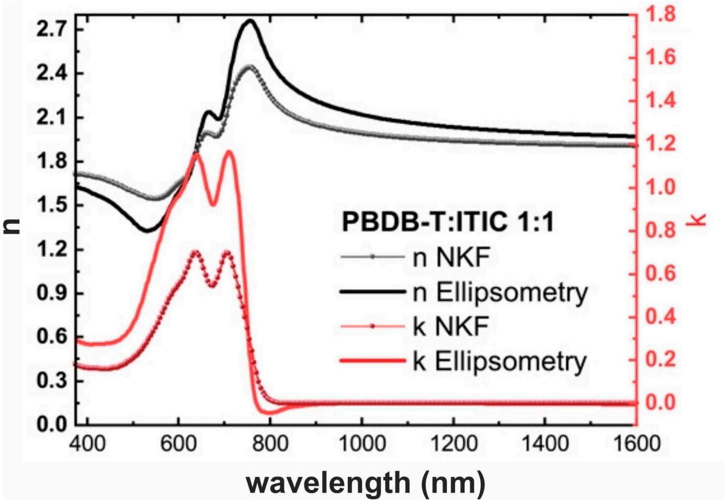

Figure S5. Optical properties of PTB7-Th:ITIC. n - refractive index, k - extinction coefficient. Data is taken from Ref.[1].

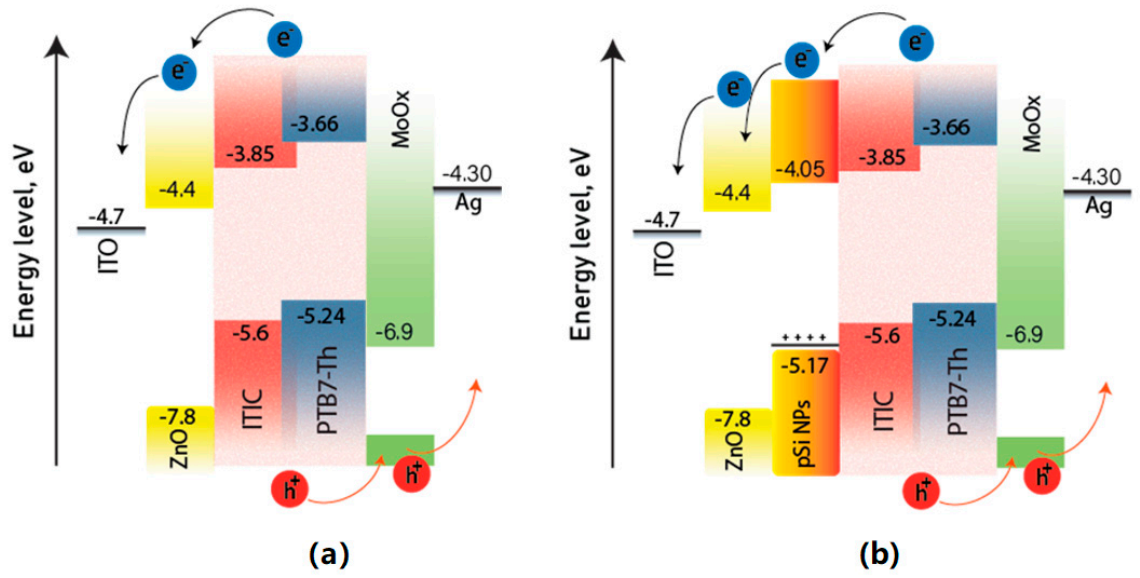

Figure S6. (a) Energy band diagram of the device without and (b) with Si NPs.

Table S1. Comparison of Photovoltaic performance enhancement of OSCs devices with plasmonic Au NPs [2] and dielectric Si NPs (our work) for the same photoactive layer.

	ΔV_{oc} V	ΔI_{sc} mA/cm^2	ΔFF %	ΔPCE relative %
ZnO:0.05 wt% Au NPs	-1.4%	+15%	+7%	+24%
Our work Si NPs with average size of 150 nm	+2%	+11%	+13%	+25%

References:

[1] Kerremans, R.; Kaiser, C.; Li, W.; Zarrabi, N.; Meredith, P.; Armin, A. The Optical Constants of Solution-Processed Semiconductors—New Challenges with Perovskites and Non-Fullerene Acceptors. Advanced Optical Materials 2020, 8(16), 2000319

[2] Usmani, B; Ranjan, R.; Prateek, Gupta, S. K.; Gupta, R.K.; Nalwa, K. S.; Garg, A.; Inverted PTB7-Th:PC71BM organic solar cells with 11.8% PCE via incorporation of gold nanoparticles in ZnO electron transport layer. Solar Energy 2021, 214, 220-230
